# Structures of the mycobacterial MmpL4 and MmpL5 transporters provide insights into their role in siderophore export and iron acquisition

**DOI:** 10.1371/journal.pbio.3002874

**Published:** 2024-10-18

**Authors:** Rakesh Maharjan, Zhemin Zhang, Philip A. Klenotic, William D. Gregor, Marios L. Tringides, Meng Cui, Georgiana E. Purdy, Edward W. Yu

**Affiliations:** 1 Department of Pharmacology, Case Western Reserve University School of Medicine, Cleveland, Ohio, United States of America; 2 Department of Pharmaceutical Sciences, Northeastern University School of Pharmacy, Boston, Massachusetts, United States of America; 3 Department of Molecular Microbiology and Immunology, Oregon Health and Science University, Portland, Oregon, United States of America; Rutgers University-Robert Wood Johnson Medical School, UNITED STATES OF AMERICA

## Abstract

The *Mycobacterium tuberculosis* (Mtb) pathogen, the causative agent of the airborne infection tuberculosis (TB), harbors a number of mycobacterial membrane protein large (MmpL) transporters. These membrane proteins can be separated into 2 distinct subclasses, where they perform important functional roles, and thus, are considered potential drug targets to combat TB. Previously, we reported both X-ray and cryo-EM structures of the MmpL3 transporter, providing high-resolution structural information for this subclass of the MmpL proteins. Currently, there is no structural information available for the subclass associated with MmpL4 and MmpL5, transporters that play a critical role in iron homeostasis of the bacterium. Here, we report cryo-EM structures of the *M*. *smegmatis* MmpL4 and MmpL5 transporters to resolutions of 2.95 Å and 3.00 Å, respectively. These structures allow us to propose a plausible pathway for siderophore translocation via these 2 transporters, an essential step for iron acquisition that enables the survival and replication of the mycobacterium.

## Abstract 2

Mycobacterium tuberculosis bacteria express several MmpL membrane protein transporters that are critical for the export of lipid components for cell wall biogenesis. These authors solve the first cryo-EM structures of the subclass I MmpL4 and MmpL5 proteins, providing insights into substrate translocation.

## Introduction

*Mycobacterium tuberculosis* (Mtb), the causative agent of the airborne infection tuberculosis (TB), is one of the deadliest human pathogens, exceeding both malaria and HIV [1,2]. Approximately 1/3 of the world’s population is infected by Mtb with most having the latent form of disease, yet approximately 10% progress to active TB [2]. The emergence and spread of multidrug-resistant (MDR)-TB compounds an increasingly difficult therapeutic challenge and severely threatens global public health. Unfortunately, TB is synergetic to both HIV [3] and COVID-19 [4] and can further escalate the severity of these infections.

The complex structure of the Mtb cell envelope plays a dominant role in the pathogenesis of the bacterium. The outer mycomembrane is very rigid and extremely impermeable to a wide range of compounds, including many antimicrobials [5]. It also provides a strong barrier against the host immune response. The outer mycomembrane is defined by an abundance of long chain mycolic acids (MAs). MAs are exported across the inner membrane of Mtb as trehalose monomycolates (TMMs) and then either covalently linked to the arabinogalactan-peptidoglycan layer as mycolyl arabinogalactan peptidoglycans (mAGPs) or incorporated into trehalose dimycolates (TDMs), which constitute the majority of the outer leaflet of this membrane. The outer leaflet also contains other non-covalently associated lipids, such as phthiocerol dimycocerosates (PDIMs) and sulfolipids (SLs) [5].

The genome of Mtb encodes 13 mycobacterial membrane protein large (MmpL) transporters [6]. These membrane proteins belong to a subfamily of the resistance-nodulation cell division (RND) superfamily of transporters [7] and are critical for mycobacterial physiology and pathogenesis, with several of these transporters involved in the export of fatty acids and lipid components to the cell envelope. MmpL3 is absolutely essential and transports TMM to the mycobacterial surface [8–10]. MmpL11 exports very long chain triacylglycerols (LC-TAG) and mycolate wax ester (MWE) [11,12]. MmpL4 and MmpL5 share similar functions in siderophore export and are required for iron acquisition [13]. MmpL7 and MmpL8 contribute to the transport of virulence-associated lipids PDIM and SL-1 [14,15], respectively, whereas MmpL10 is involved in the translocation of diacyltrehalose to the cell envelope [16]. Besides the essential gene *mmpL3* in Mtb, it has been shown that disruption of *mmpL4*, *mmpL5*, *mmpL7*, *mmpL8*, *mmpL10*, and *mmpL11* lead to significant virulence attenuation in a mouse model [8,17–20].

In addition to exporting important lipid elements for cell wall biogenesis, select MmpL transporters have been reported to participate in the active efflux of anti-TB drugs. For example, up-regulation of MmpL5 confers increased resistance to bedaquiline [21], an FDA approved anti-TB drug, and MmpL7 contributes to isoniazid and ethionamide resistance when overexpressed [22]. Currently, MmpL3 is considered an important pharmacologic target for anti-TB drug discovery [23], making it a high priority to elucidate the molecular mechanisms of this transporter.

The phylogenetic tree reveals that MmpL proteins can be separated into 2 distinct subclasses [24]. The majority of MmpLs, such as MmpL4, MmpL5, MmpL7, MmpL8, and MmpL10, belong to subclass I. It is anticipated that MmpL proteins in this subclass contain only 2 domains: a transmembrane domain and a periplasmic domain [24]. However, proteins within subclass II of the MmpL subfamily, including MmpL3 and MmpL11, are predicted to possess transmembrane and periplasmic domains with an additional C-terminal cytoplasmic domain [24] (S1 Fig).

We and others have previously solved high-resolution structures of the MmpL3 transporter from *M*. *smegmatis* and *M*. *tuberculosis* using either X-ray crystallography or cryo-electron microscopy (cryo-EM) [25–31]. Importantly, we also determined the structures of MmpL3 bound with the lipid TMM [26]. These structures revealed that MmpL3 consists of at least 2 TMM lipid binding sites that promote the transport of lipids across the cytoplasmic membrane, allowing us to propose a mechanism of TMM translocation for cell wall biosynthesis [25,26].

Thus far, there is no structural information of MmpL transporters that are affiliated with subclass I. To facilitate modeling protein structures of these important membrane proteins, we here present cryo-EM structures of the *M*. *smegmatis* MmpL4 and MmpL5 transporters to resolutions of 2.95 Å and 3.00 Å, respectively. We observed that both MmpL4 and MmpL5 form a channel that spans from the outer leaflet of the cytoplasmic transmembrane domain through the periplasmic domain, suggesting a plausible pathway for substrate translocation.

## Results and discussion

### Structure of the *M*. *smegmatis* MmpL4 transporter

**Fig 1:**
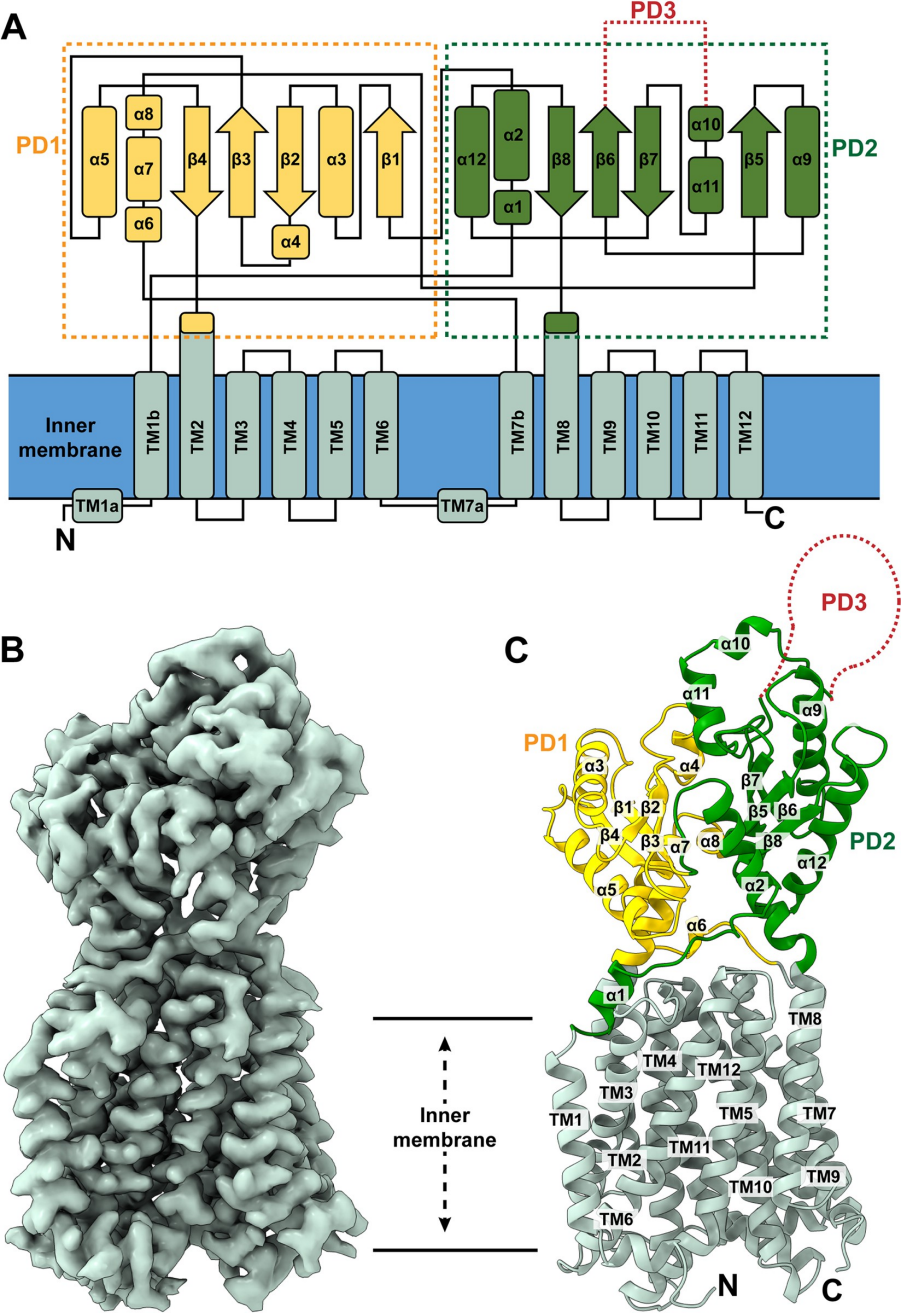
Structure of the *M*. *smegmatis* MmpL4 transporter. (A) Secondary structural topology of MmpL4. The topology was constructed based on the cryo-EM structure of MmpL4. The transmembrane helices (TMs) are colored pale green. The secondary structural elements of the periplasmic subdomains PD1 and PD2 are colored yellow and green, respectively. The missing subdomain PD3 is represented by red dotted lines. (B) Cryo-EM map of *M*. *smegmatis* MmpL4 at a resolution of 2.95 Å. (C) Ribbon diagram of the structure of MmpL4 viewed in the membrane plane. The secondary structural elements of the TM domain, PD1 subdomain, and PD2 subdomain are colored pale green, yellow, and green, respectively. The missing PD3 subdomain is represented by a red dotted curve. The PDB and EMDB accession codes of the structure of MmpL4 are 9B43 and EMD-44167, respectively. cryo-EM, cryo-electron microscopy; MmpL, mycobacterial membrane protein large.

We were particularly interested in determining the structures of the MmpL4 and MmpL5 transporters, as they have been shown to be critical in the iron acquisition pathway of the mycobacterium [32]. Mycobacteria secrete mycobactin (Mbt) and carboxymycobactin (Cmb), siderophores with an extremely high affinity for Fe(III), via the MmpL4 and MmpL5 transporters to recruit this important metal that is essential for growth and survival [13]. We cloned the *M*. *smegmatis* full-length MmpL4 transporter (969 amino acids) into the *Escherichia coli* expression vector pET15b with a 6xHis tag at the C-terminus. This MmpL4 membrane protein was overproduced in *E*. *coli* BL21(DE3)*ΔacrB* cells and purified using Ni^2+^-affinity and Superdex 200 size exclusion columns. We then collected single-particle cryo-EM images of the MmpL4 transporter embedded in LMNG detergent micelles and solved its structure to a resolution of 2.95 Å (Figs 1 and S2 and S1 Table).

Our cryo-EM structure indicates that MmpL4 is monomeric in form with overall dimensions of 110 Å × 75 Å × 55 Å. This transporter consists of a large membrane-spanning domain formed by 12 transmembrane helices (TMs 1–12) and a large periplasmic domain formed by 2 hydrophilic loops (loops 1 and 2), consistent with the signature of the RND superfamily of proteins [7] (Fig 1A and 1B). Periplasmic loop 1 is located between TMs 1 and 2, whereas loop 2 is situated between TMs 7 and 8. These 2 loops coordinate and contribute to create the periplasmic subdomains PD1 and PD2. It should be noted that our cryo-EM structure contains most MmpL4 residues except for residues 495–689, which presumably form the PD3 subdomain located right above subdomain PD2.

Interestingly, there appears to be a resemblance in the overall structure of MmpL4 to that of MmpL3, although MmpL3 and MmpL4 only share 22% protein sequence identity. These 2 MmpL transporters are superimposable; however, superimposition of the TM domain, and PD1 and PD2 subdomains of the structure of MmpL4 to those of MmpL3 (PDB ID: 7K8B) [26] gives rise to a very high root mean square deviation (RMSD) of 5.9 Å (for 673 Cα atoms), indicating the structures of MmpL3 and MmpL4 are very distinct from each other (S3 Fig).

The N-terminal and C-terminal halves of MmpL4 are structurally related to each other by a pseudo 2-fold symmetry, but these 2 halves share a low protein sequence identity of only 16%. These 2 halves can be superimposed, but the RMSD is quite high for this superimposition (2.6 Å for 294 Cα atoms) (S3 Fig). It should be noted that a similar high RMSD of 2.6 Å has also been found when the 2 halves of the MmpL3 transporter are superimposed [25]. PD1 consists of 6 α-helices (α3, α4, α5, α6, α7, and α8), 4 β-strands (β1, β2, β3, and β4), and the first 10 N-terminal residues of TM2. The majority of the PD1 amino acids originate from loop 1. However, helix α7 (428–437) of loop 2 also participates in forming this subdomain. PD2 is composed of 6 α-helices (α1, α2, α9, α10, α11, and α12), 4 β-strands (β5, β6, β7, and β8), and the first 10 N-terminal residues of TM8. The PD2 amino acids mainly originate from loop 2, but helix α2 (residues 72–83) of loop 1 also contributes to this periplasmic subdomain. The crossover of these 2 loops allows the 2 periplasmic subdomains to connect with each other in the periplasm. Notably, the periplasmic domains of MmpL4 are presumed to be flexible in nature as evidenced by the existence of long linkers. Residues 52–71 are predominantly unstructured and create a long linker connecting the C-terminus of TM1 of the transmembrane domain and the N-terminus of helix α2 of the PD2 periplasmic subdomain, although residues 55–61 form helix α1. Likewise, residues 413–427, which also include helix α6 (419–422), form a linker bridging the C-terminal end of TM7b and the N-terminal end of helix α7 of PD1 together. The TMs of MmpL4 are membrane embedded, but both TM2 and TM8 are significantly longer and protrude into the periplasmic region to participate in creating PD1 and PD2, respectively, similar to what is observed in the structures of MmpL3 [25–31]. These 2 TMs directly tether the 2 periplasmic subdomains and form part of the periplasmic structure of the protein. It should be noted that residues 495–689 are missing in our MmpL4 structure. Based on the structural information, these residues should form an independent periplasmic subdomain PD3, which may be critical for interacting with other periplasmic accessory proteins, such as mycobacterial membrane protein small 4 (MmpS4) and other periplasmic adaptor proteins including Rv0455c [33]. It should be noted that our MmpL4 protein is a full-length protein as indicated by size exclusion chromatography (SEC) and SDS-PAGE of the purified protein (S4 Fig). It is likely that subdomain PD3 is highly flexible, evidenced by the fact that we did not observe any cryo-EM densities arising from this subdomain even though the contour level of the map was set to a very low threshold (S4 Fig).

### Structure of the *M*. *smegmatis* MmpL5 transporter

**Fig 2:**
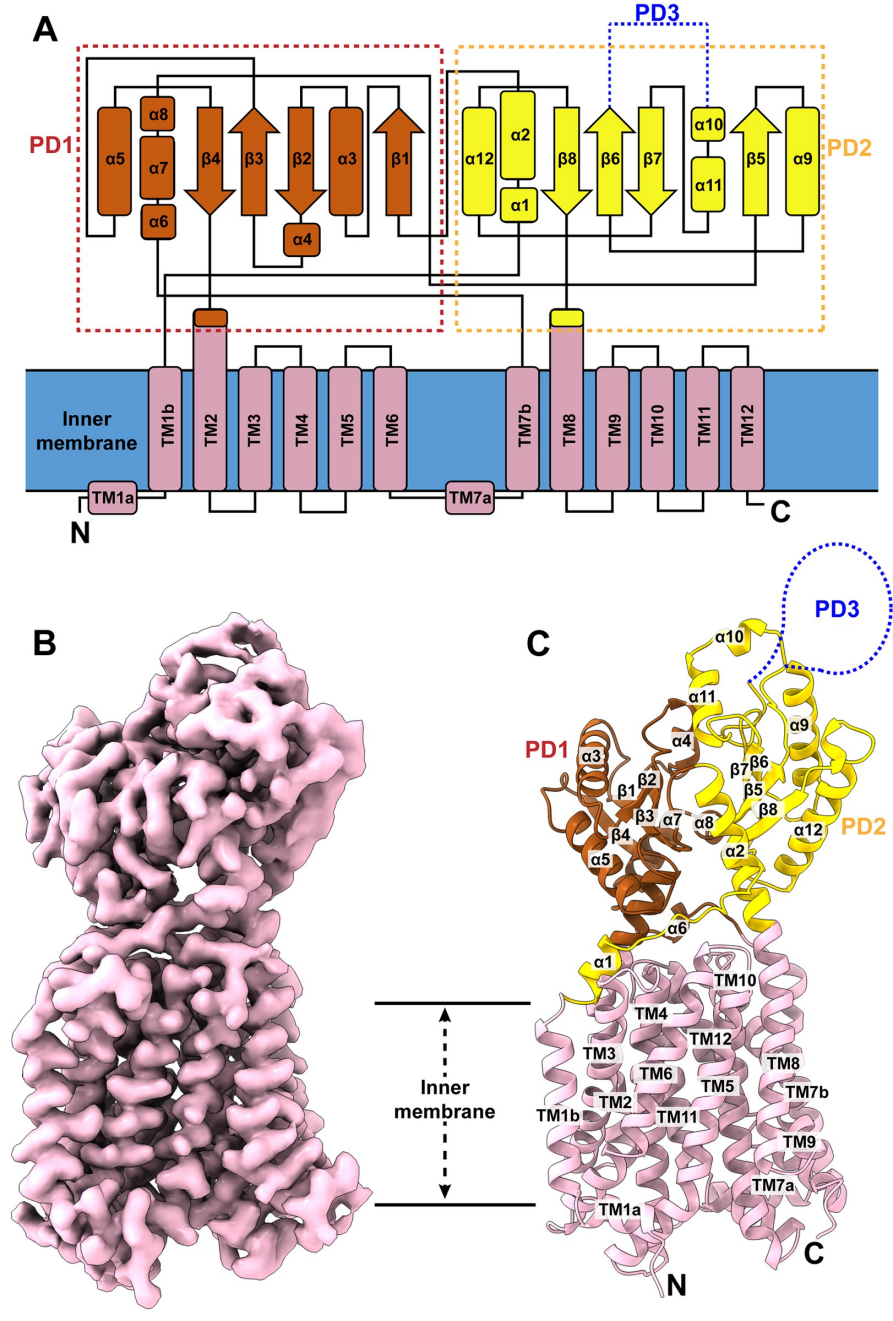
Structure of the1 *M*. *smegmatis* MmpL5 transporter. (A) Secondary structural topology of MmpL5. The topology was constructed based on the cryo-EM structure of MmpL5. The transmembrane helices (TMs) are colored pink. The secondary structural elements of the periplasmic subdomains PD1 and PD2 are colored brown and yellow, respectively. The missing subdomain PD3 is represented by blue dotted lines. (B) Cryo-EM map of *M*. *smegmatis* MmpL5 at a resolution of 3.00 Å. (C) Ribbon diagram of the structure of MmpL5 viewed in the membrane plane. The secondary structural elements of the TM domain, PD1 subdomain, and PD2 subdomain are colored pink, brown, and yellow, respectively. The missing PD3 subdomain is represented by a blue dotted curve. The PDB and EMDB accession codes of the structure of MmpL5 are 9B46 and EMD-44171, respectively. cryo-EM, cryo-electron microscopy; MmpL, mycobacterial membrane protein large.

MmpL5 is homologous to MmpL4. These 2 membrane proteins share 65% protein sequence identity, perform similar functions in siderophore export and are required for iron acquisition [13,34]. However, only MmpL5 appears to confer increased resistance to bedaquiline [21], an FDA approved anti-TB drug. As the structure of MmpL4 is missing the periplasmic subdomain PD3 of approximately 200 residues, we thought that the structure of MmpL5 could provide the structural information of this missing subdomain. We therefore used a similar approach to clone the full-length *M*. *smegmatis* MmpL5 transporter into the vector pET15b with a 6xHis tag at the C-terminus to generate pET15bΩ*mmpL5*. This MmpL5 membrane protein was overproduced in *E*. *coli* BL21(DE3)*ΔacrB* cells and purified using Ni^2+^-affinity and Superdex 200 size exclusion columns. The cryo-EM structure of MmpL5 embedded in LMNG detergent micelles was solved to a nominal resolution of 3.00 Å (Figs 2 and S5 and S1 Table).

The cryo-EM structure of MmpL5 is nearly identical to that of MmpL4. Superimposition of these 2 structures gives rise to an RMSD of 0.9 Å (for 681 Cα atoms). Like MmpL4, TM1-TM12 of the transmembrane domain and PD1-PD2 of the periplasmic domain are clearly defined in the cryo-EM map. However, cryo-EM densities for the majority of residues making up the periplasmic subdomain PD3 are missing, similar to what was seen with MmpL4. This result adds additional evidence that PD3 is highly flexible, which may allow this subdomain to have a higher degree of freedom for finding binding partners in the periplasm.

PD1 is composed of 6 α-helices and 4 β-strands, including α3, α4, α5, α6, α7, α8, β1, β2, β3, and β4. The majority of these structural elements belong to periplasmic loop 1 of MmpL5, but helix α7 of loop 2 also contributes to form this subdomain. PD2 contains 6 α-helices and 4 β-strands, which include α1, α2, α9, α10, α11, α12, β5, β6, β7, and β8. Most of these structural elements, except helix α2, arise from loop 2 of the protein sequence. Like MmpL4, the periplasmic domain of MmpL5 possesses several linkers to tether these 2 periplasmic subdomains together as well as attach them to the transmembrane domain. These findings indeed highlight the flexible nature of the MmpL5 transporter.

### The MmpL4 and MmpL5 channels

Within the structure of MmpL3, a channel-like cavity spanning the outer leaflet of the inner membrane up through the periplasmic domain was observed. Interestingly, it was previously found that MmpL3 possesses 2 TMM lipid-binding sites [35]. The first bound TMM molecule was located within the pocket at the outer leaflet of the cytoplasmic membrane. This binding pocket, which forms the beginning of the elongated channel, is created by TMs 7–10 of the transmembrane region of MmpL3. The second bound TMM lipid was found to sandwich in a cavity generated between subdomains PD1 and PD2 of the periplasmic domain of MmpL3. This cavity is situated within the passageway of the MmpL3 channel. Coupled together, the structural data and computational simulations have led to a proposed mechanism for substrate translocation, where the MmpL3 transporter exports TMM lipids from the outer leaflet of the transmembrane up through the periplasm [35].

**Fig 3:**
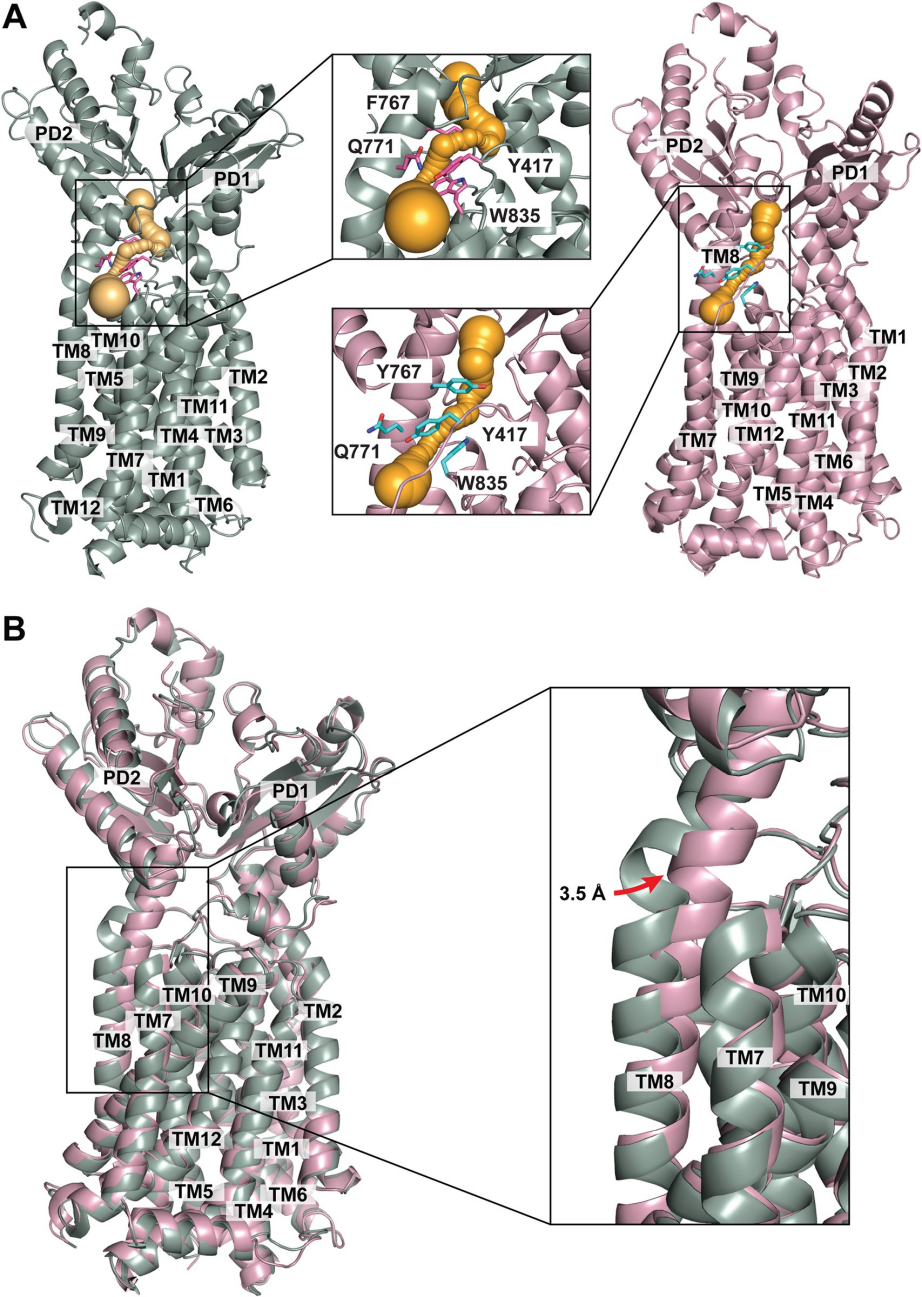
Structural comparison of the MmpL4 and MmpL5 transporters. (A) Channel formation in each MmpL transporter. The MmpL4 protomer (green) forms a channel (orange) spanning the outer leaflet of the inner membrane up through the periplasmic domain. Residues Y417, Y767, Q771, and W835 (magenta sticks) create the narrowest region of this MmpL4 channel. Similarly, the MmpL5 protomer (pink) forms a channel (orange) spanning the outer leaflet of the inner membrane up through the periplasmic domain. Residues Y417, Y767, Q771, and W835 (cyan sticks) create the narrowest region of this MmpL5 channel. (B) Superimposition of the structures of the MmpL4 and MmpL5 transporters. This superimposition indicates that the major conformational difference between MmpL4 and MmpL5 is the location of TM8. It appears that the section of TM8 located at the outermost surface of the cytoplasmic membrane of MmpL5 shifts into the core by as much as 3.5 Å in relation to the position of TM8 in MmpL4. This rigid-body shift may help control the opening and closing of both channels formed within the MmpL4 and MmpL5 transporters. MmpL, mycobacterial membrane protein large.

We used the program CAVER (https//www.caver.cz/) to identify channel formation in MmpL4. Similar to MmpL3, the structure of the MmpL4 transporter also forms an elongated channel spanning the pocket surrounded by TMs 7–10 at the outer leaflet of the cytoplasmic membrane and the cavity generated between subdomains PD1 and PD2 in the periplasm (Fig 3A). The pocket located at the transmembrane domain and the cavity sandwiched between the 2 periplasmic subdomains potentially harbors substrate binding sites for anchoring siderophore substrates such as Mbts. This elongated channel is continuously open and directly connects these 2 potential siderophore-binding sites. Therefore, this conformation of MmpL4 may represent the open-channel state of this transporter, and this channel is likely used to export siderophore substrates. Additionally, both the pocket surrounded by TMs 7–10 and the cavity between PD1 and PD2 can bind Mbt in the micromolar range based on docking calculations (see below). It should be noted that we also observed a channel with an opening orchestrated by TMs 1–4 of MmpL4 (S6 Fig). However, this pocket is not capable of binding Mbt based on docking calculations (see below). Therefore, it is unlikely that the pocket surrounded by TMs 1–4 can form an entrance for siderophore transport.

We used the same approach, utilizing the program CAVER (https//www.caver.cz/), to search for channel formation in the MmpL5 transporter. Distinct from MmpL4, the structure of MmpL5 indicates that this transporter only possesses one channel that spans the outer leaflet of the transmembrane domain up through the periplasmic domain (Fig 3A). However, the midway point of this channel is much narrower, suggesting that the structure of MmpL5 may depict a different intermediate state with respect to that of MmpL4.

In comparing the structures of MmpL4 and MmpL5, it can be interpreted that TM8 is critically important for the opening and closing of the transporter’s channel (Fig 3B). In MmpL5, the section of TM8 located at the outermost surface of the cytoplasmic membrane shifts into the core of the membrane protein by as much as 3.5 Å in relation to the position of TM8 in MmpL4 (Fig 3B). This rigid-body shift may help control the opening and closing of the channels formed within the MmpL4 and MmpL5 transporters. In MmpL5, the narrowest region of the channel is surrounded by residues Y417, Y767, Q771, and W835, residues that may be important for controlling the width of the MmpL5 channel (Fig 3A). The corresponding 4 residues in MmpL4 (Y417, F767, Q771, and W835) also create the narrowest region of its channel (Fig 3A). Therefore, in both instances, these residues appear critical for controlling the opening and closing of the channel, thus regulating substrate export.

### The MmpL4 and MmpL5 proton-relay networks

According to the protein sequence alignment with MmpL3, it is speculated that both MmpL4 and MmpL5 are proton motive force (PMF)-dependent transporters that function via a proton/substrate antiport mechanism. Within the transmembrane domain of MmpL3, the conserved residues D256, Y257, D645, and Y646 are involved in forming the proton-relay network for energy coupling [25,27] (S7 Fig). Interestingly, these proton-relay residues are conserved between MmpL3 and MmpL4. The corresponding residues in MmpL4 are D278, Y279, D851, and Y852 (S7 Fig). Based on the structural information of MmpL4, residue D278 forms a hydrogen bond with Y852, presumably enabling the transfer of protons to facilitate substrate transport. Within the transmembrane domain of the MmpL5 transporter, the conserved residues that make up the proton-relay network are also residues D278, Y279, D851, and Y852 (S7 Fig). Similar to MmpL4, residue D278 and Y852 of MmpL5 interact with each other and create a hydrogen bond, presumably relaying protons for energy coupling.

### Docking of mycobactin onto MmpL4 and MmpL5

MmpL3 binds TMM in the pocket formed by TM7-10 and the cavity located between PD1 and PD2. We observed that MmpL4 also possesses a similar pocket and cavity that potentially generate 2 siderophore-binding sites. Several hydrophobic and polar residues, including L407, L410, Y413, F334, A774, N775, L778, I779, V811, I812, G815, F818, W835, L838, A839, and V842, surround the pocket formed by TM7-10 of the MmpL4 transporter. Many of these residues belong to TM8, TM9, and TM10, and these lining residues may constitute a binding site for siderophores. Interestingly, it appears that TM8 and TM9 coordinate to form a V-shape feature to create part of this pocket. At the periplasmic domain of MmpL4, a mixture of hydrophobic, polar, and charged residues line the wall of the periplasmic central cavity sandwiched between PD1 and PD2. These residues include M66, M77, N90, S92, Q159, P191, L194, S195, Q198, F263, Y333, N418, D419, R420, Y433, A443, N446, P447, D727, P728, M729, T763, T766, F767, H834, and M836 and may constitute a second siderophore-binding site. In view of this central cavity, residues of the 2 extended loops that run across subdomains PD1 and PD2 (residues 83–92 and residues 437–448) form the front and back sides of this cavity in relation to the orientation of Fig 1. Residues 61–72 of the loop connecting TM1 and PD2, and residues 416–426 of the elongated loop connecting TM7 and PD1 form the bottom of this large cavity. Additionally, the periplasmic loop of PD1 (residues 155–162), the periplasmic loop of PD2 (residues 724–731), the N-terminal end of TM2 (residues 191–195), and the N-terminal end of TM7 (residues 416–426) surround different sides to secure the formation of this cavity. It should be noted that TM8 participates in forming both the pocket at the membrane region and the periplasmic central cavity. Therefore, TM8 may be required for substrate recognition and possibly substrate transport.

**Fig 4:**
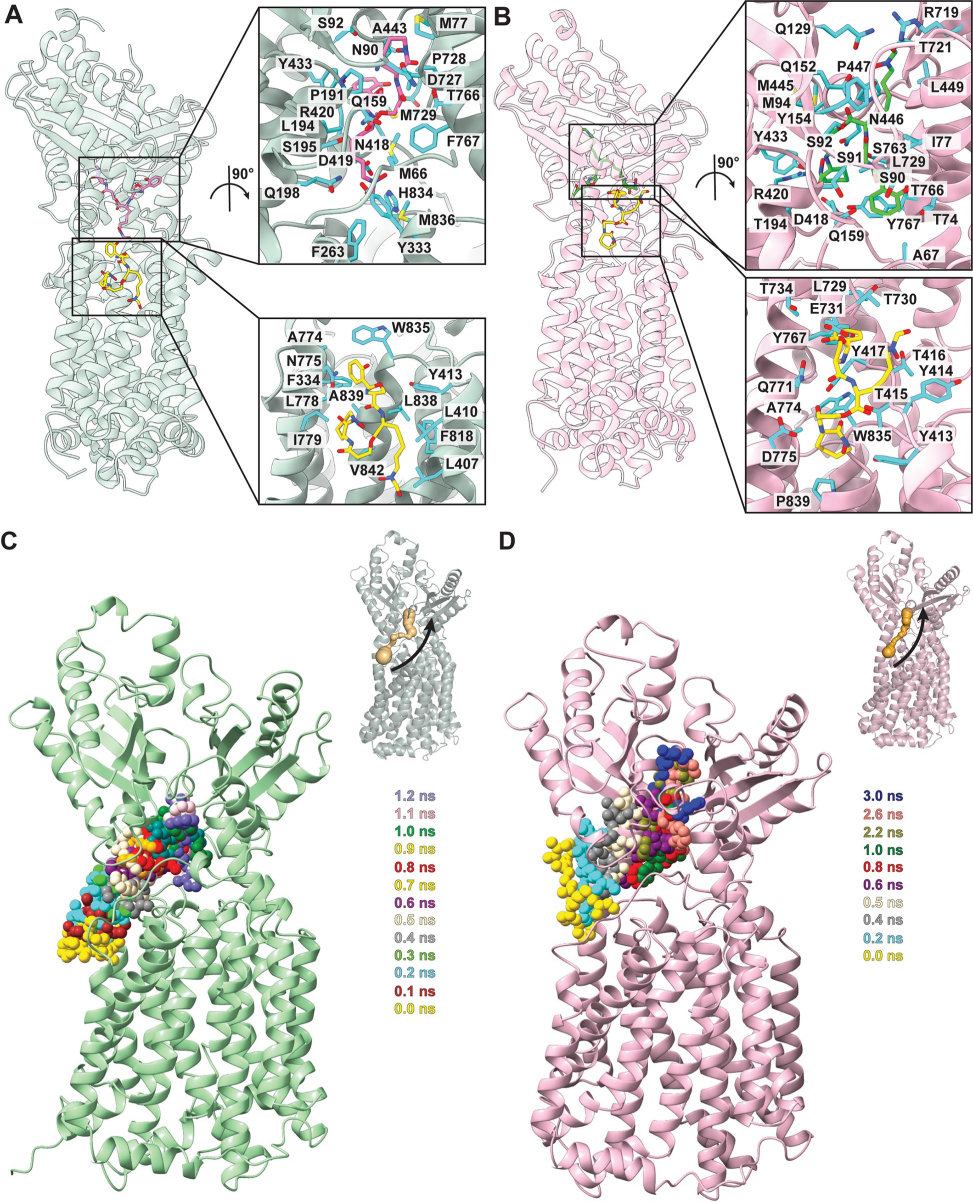
Docking of Mbt and targeted MD simulation. (A) The predicted Mbt binding sites of MmpL4. The docked Mbt molecules are shown as yellow sticks (at the binding site located in the outer leaflet of the cytoplasmic membrane) and magenta sticks (at the binding site located in periplasmic domain). Residues predicted to be responsible for Mbt binding at both binding sites are in cyan sticks. (B) The predicted Mbt binding sites of MmpL5. The docked Mbt molecules are shown as yellow sticks (at the binding site located in the outer leaflet of the cytoplasmic membrane) and green sticks (at the binding site located in periplasmic domain). Residues predicted to be responsible for Mbt binding at both binding sites are shown as cyan sticks. (C) Targeted MD simulations showing different time points for MmpL4–Mbt interactions (0 ns to 1.2 ns). (D) Targeted MD simulations showing different time points for MmpL5-Mbt interactions (0 ns to 3.0 ns). Mbt, mycobactin; MD, molecular dynamics; MmpL, mycobacterial membrane protein large.

To predict how MmpL4 recognizes and contacts siderophore substrates, we used AutoDock Vina [36] to computationally dock the mycobactin (Mbt) ligand onto MmpL4. The docking predictions suggest that MmpL4 forms 2 siderophore-binding sites. The locations of these bound Mbt compounds overlap with the pocket and cavity constituted within the transmembrane and periplasmic regions of the transporter (Fig 4A). The predicted Mbt binding affinities are −7.1 and −5.4 kcal/mol for the transmembrane and periplasmic binding sites of MmpL4, respectively. These binding affinities roughly correspond to dissociation constants (K_D_s) of 6.1 and 92 μm. These values are in the same range as those found in MmpL3, where MmpL3 binds TMM and phosphatidylethanolamine (PE) lipids with measured K_D_s of 3.7 and 19.5 μm, respectively [25]. Interestingly, these binding strengths are also comparable with those of many other RND-type multidrug efflux pumps, where these pumps bind toxic compounds and antibiotics within the micromolar range [37–40].

We also analyzed how MmpL5 potentially interacts with Mbt. The transmembrane binding pocket of MmpL5 consists of several residues, including Y413, Y414, T415, T416, Y417, L729, T730, E731, T734, Y767, Q771, A774, D775, L838, and P839. Again, many of these residues originate from TM8, TM9, and TM10. The periplasmic central binding cavity of MmpL5 contains various residues, such as A67, T74, I77, S90, S91, S92, M94, Q129, Q152, Y154, Q159, T194, D418, R420, Y433, M445, N446, L449, R719, T721, T766, and Y767, which are hydrophobic, polar, or charged in nature (Fig 4B). This cavity is surrounded by several loops as well as the N-terminal residues of TM2 and the N-terminal residues of TM8, similar to what is observed in the MmpL4 periplasmic cavity. Autodock Vina suggests that MmpL5 binds Mbt in the transmembrane pocket and periplasmic cavity with affinities of −7.9 and −8.0 kcal/mol, which correspond to K_D_s of 1.6 and 1.3 μm, respectively. Again, these strengths are in the same range as those found in MmpL3–lipid interactions [25].

We also used Vina [36] to predict potential substrate binding sites for carboxymycobactin (Cmb) and bedaquiline (Bed) in the MmpL4 and MmpL5 transporters. For MmpL4, the results indicate that MmpL4 possesses 1 Cmb binding site with a predicted binding affinity of −7.0 kcal/mol. This site is located at the outer leaflet of the transmembrane domain and is surrounded by TMs 7–10 (S8A Fig). MmpL4 also possesses 1 Bed binding site with a predicted binding affinity of −7.1 kcal/mol, although increased Bed resistance due to MmpL4 has not been reported. This site coincides with that of the Cmb binding site (S8B Fig). In the case of MmpL5, this transporter appears to contain 2 Cmb binding sites and the locations of these 2 sites are similar to those found for the Mbt binding sites, at the outer leaflet of the transmembrane domain and at the periplasmic domain. The predicted binding affinities for these 2 sites are −6.9 kcal/mol and −7.4 kcal/mol, respectively (S8C Fig). However, MmpL5 only contains 1 Bed binding site with a predicted binding affinity of −8.4 kcal/mol. This site is located at the outer leaflet of the transmembrane domain and is surrounded by TMs 7–10 (S8D Fig).

### Computational simulations of the MmpL4 and MmpL5 transporters

Based on the docking data for MmpL4 and MmpL5, there are 2 potential siderophore-binding sites for these membrane proteins. In both MmpL4 and MmpL5, one of these binding sites is located at the pocket surrounded by TMs 7–10 while the other is within the large cavity between the periplasmic PD1 and PD2 subdomains. To further elucidate the mechanism of siderophore transport, we used the cryo-EM structure of MmpL4 and utilized the NAMD program [41] to perform targeted molecular dynamics (MD) simulations. We chose the substrate Mbt as docking experiments indicate that this siderophore is bound by the MmpL4 transporter. We observed that Mbt is anchored at the siderophore-binding site formed between TMs 7–10 (Fig 4C). During the process of Mbt transport to the periplasmic domain, the narrowest region of the MmpL4 channel formed by the N-terminal end of TM8 (residues 763–774), the flexible loop connecting TM7 and helix α6 (residues 413–419), and the flexible loop between β7 and helix α12 (residues 724–730) is found to gradually open in order to allow Mbt to reach the periplasmic cavity formed between subdomains PD1 and PD2. According to targeted MD simulations, the opening and closing of the MmpL4 channel is accompanied by a change in conformation of the first 12 N-terminal amino acids of TM8, where residues 763–774 of TM8 swing towards the phospholipid bilayer of the cytoplasmic membrane and away from the central core to facilitate channel opening. This observation is in good agreement with the structural comparison of MmpL4 and MmpL5, where channel opening and closing may be controlled by the rigid-body movement of TM8 near the periplasmic surface of the cytoplasmic membrane.

We also performed targeted MD simulations on the MmpL5 transporter with Mbt as the transported substrate (Fig 4D). We observed that Mbt is bound within the siderophore-binding site between TMs 7–10. Similar to MmpL4, the narrowest region of the MmpL5 channel is formed by the N-terminal end of TM8 (residues 763–774), the flexible loop connecting TM7 and helix α6 (residues 413–419), and the flexible loop between β7 and helix α12 (residues 724–730) is found to gradually open to permit Mbt to reach the periplasmic domain, between PD1 and PD2, of MmpL5.

Based on targeted MD simulations, it is likely that both MmpL4 and MmpL5 shuttle siderophores from the outer leaflet of the cytoplasmic membrane via the substrate binding site formed by TMs 7–10. These siderophore molecules are then transferred to the periplasmic binding cavities of MmpL4 and MmpL5 via the channels created by these 2 transporters (S9 Fig). In each transporter, it appears that TM8 is critically important to control the opening and closing of the channel, allowing it to propel its siderophore substrates from the outer leaflet of the cytoplasmic membrane to the periplasm. Previously, it was found that MmpL3 is a flippase [42] capable of translocating lipid elements from the inner leaflet to the outer leaflet of the cytoplasmic membrane. There is a chance that both the MmpL4 and MmpL5 transporters may also behave like flippases. If this is the case, then these 2 transporters may also be able to shuttle siderophores from the inner leaflet of the cytoplasmic membrane to the periplasm of the mycobacterium.

## Methods

### Expression and purification of MmpL4 and MmpL5

The *M*. *smegmatis* MmpL4 gene (MSMEG_3496) was cloned into the pET15bΩ*mmpL4* expression vector in frame with a 6xHis tag at the C-terminus. This tagged MmpL4 protein was overproduced in *Escherichia coli* BL21(DE3)*ΔacrB* cells, which harbor a deletion in the chromosomal *acrB* gene. Cells were grown in 12 L of LB medium with 100 μg/ml ampicillin at 37°C. When the OD_600 nm_ reached 0.4, the culture was treated with 0.2 mM isopropyl-β-D-thiogalactopyranoside (IPTG) to induce *mmpL4* expression at 37°C. Cells were then harvested within 4 h of induction. The collected bacteria were resuspended in low salt buffer (100 mM sodium phosphate (pH 7.2), 10% glycerol, 1 mM ethylenediaminetetraacetic acid (EDTA), and 1 mM phenylmethylsulfonyl fluoride (PMSF)) and then disrupted with a French pressure cell. The membrane fraction was collected and washed twice with high salt buffer (20 mM sodium phosphate (pH 7.2), 2 M KCl, 10% glycerol, 1 mM EDTA, and 1 mM PMSF), and once with 20 mM HEPES-NaOH buffer (pH 7.5) containing 1 mM PMSF as described previously [43]. The membrane protein was then solubilized in 1% (w/v) lauryl maltose neopentyl glycol (LMNG) overnight at 4°C. Insoluble material was removed by ultracentrifugation at 100,000 × g. The extracted protein was then purified with a Ni^2+^-affinity column. The purified protein was dialyzed against 20 mM Na-HEPES (pH 7.5) and concentrated in a buffer containing 20 mM Na-HEPES (pH 7.5) and 0.01% LMNG. A final purification step was performed using a Superdex 200 size exclusion column loaded with buffer containing 20 mM Na-HEPES (pH 7.5) and 0.005% LMNG. The purity of the MmpL4 protein (>95%) was judged using SDS-PAGE stained with Coomassie brilliant blue. The purified protein was then concentrated a second time to 10 mg/ml in a buffer containing 20 mM Na-HEPES (pH 7.5) and 0.005% LMNG.

Similarly, the *M*. *smegmatis* MmpL5 gene (MSMEG_1382) was cloned into the pET15bΩ*mmpL5* expression vector in frame with a 6xHis tag at the C-terminus. This tagged MmpL5 protein was also overproduced in *Escherichia coli* BL21(DE3)*ΔacrB* cells. The procedures used for the expression and purification of the MmpL5 protein are identical to those of MmpL4 detailed above. The purity of the MmpL5 protein (>95%) was judged using SDS-PAGE stained with Coomassie brilliant blue. The purified protein was then concentrated to 10 mg/ml in a buffer containing 20 mM Na-HEPES (pH 7.5) and 0.005% LMNG.

### Cryo-EM sample preparation

For imaging MmpL4, a 2.5 μl of 2.0 mg/ml MmpL4 sample was directly applied to glow-discharged holey carbon grids (Quantifoil Cu R1.2/1.3, 300 mesh), blotted for 10 s and then plunge-frozen in liquid ethane using a Vitrobot (Thermo Fisher). Similarly, for imaging MmpL5, a 2.5 μl of 2.0 mg/ml MmpL5 sample was applied to glow-discharged holey carbon grids (Quantifoil Cu R1.2/1.3, 300 mesh), blotted for 10 s and then plunge-frozen in liquid ethane using a Vitrobot (Thermo Fisher). All grids were then transferred into cartridges prior to data collection.

### Data collection

For the MmpL4 sample, the images were collected in super-resolution mode at 81 K magnification on a Titan Krios equipped with a K3 direct electron detector (Gatan). The physical pixel size was 1.07 Å/pix (super-resolution of 0.535 Å/pix). Each micrograph of MmpL4 or MmpL5 was exposed to a total dose of 40 e^-^/Å^2^ (defocus range of −0.8 to −1.5 μm) and 40 frames were captured using SerialEM [44].

### Data processing

For MmpL4, the super-resolution image stack was aligned and binned by 2 using patch motion. The contrast transfer function (CTF) was estimated using patch CTF in cryoSPARC [45]. Procedures for blob picker followed by 2D classification were applied to generate templates for automated template picking. Initially, 4,352,328 particles were selected after autopicking in cryoSPARC [45]. Several iterative rounds of 2D classification followed by ab initio and heterogeneous 3D classification were performed to remove false picks and classes with unclear features, ice contamination, or carbon. The resulting 78,169 particles were subjected to non-uniform refinement followed by local refinement, which led to a 2.95 Å resolution cryo-EM map of MmpL4 based on the gold standard Fourier shell correlation (FSC 0.143) (S2 Fig).

For MmpL5, the same procedure was used to generate templates for automated template picking. Initially, 1,648,768 particles were selected after autopicking in cryoSPARC [45]. Several iterative rounds of 2D classification, ab initio and heterogeneous 3D classification were performed to remove false picks and classes with unclear features. The resulting 61,169 particles were subjected to non-uniform refinement followed by local refinement with non-uniform sampling resulted in a 3.00 Å resolution cryo-EM map for MmpL5 based on the gold standard Fourier shell correlation (FSC 0.143) (S5 Fig).

### Model building and refinement

Model building of both MmpL4 and MmpL5 were based on their respective cryo-EM maps. We first generated the predicted MmpL4 and MmpL5 models, each based on the MmpL3 cryo-EM structure (PDB ID: 7K8B) [26] as the template, using the SWISS-MODEL program [46]. These predicted MmpL4 and MmpL5 structures were used and fitted into the corresponding density maps using Chimera [47]. The subsequent procedures for model rebuilding were performed using Coot [48]. Structural refinements were accomplished using the phenix.real_space_refine program [49] from the PHENIX suite [50]. The final atomic models of MmpL4 and mmpL5 were evaluated using MolProbity [51]. The statistics associated with data collection, 3D reconstruction, and model refinement are included in S1 Table.

### Molecular docking calculations

The program AutoDock Vina [36] was used to predict the Mbt-, Cmb-, and Bed-binding modes of MmpL4 and MmpL5. The structures of MmpL4 and MmpL5 were used for these docking calculations. In each calculation, the full-length MmpL4 or MmpL5 was set as a rigid structure, allowing for the search of potential substrate (Mtb, Cmb, or Bed)-binding sites throughout the entire length of the protein. However, the conformation of the Mtb, Cmb, or Bed molecule was optimized via all modeling and docking procedures. For Mbt, the calculations indicate that both MmpL4 and MmpL5 contain 2 Mbt-binding sites (at the periplasmic domain and at the outer leaflet of the transmembrane domain). For Cmb, the docking results show that MmpL4 only possesses 1 Cmb-binding site (at the outer leaflet of the transmembrane domain) but MmpL5 contains 2 Cmb-binding sites (at the periplasmic domain and at the outer leaflet of the transmembrane domain), similar to those found for Mbt. For Bed docking, the data indicate that both MmpL4 and MmpL5 contain 1 Bed-binding site (at the outer leaflet of the transmembrane domain).

### Targeted MD simulations

Targeted MD (TMD) simulations were performed using the NAMD program [41]. We used our cryo-EM structures of MmpL4 and MmpL5 for the simulations. These 2 structures were separately immersed in an explicit lipid bilayer consisting of POPC and POPE with a molecular ratio of 1:1, and a water box with dimensions of 90.2 Å × 90.2 Å × 153.3 Å (118,239 atoms) and 90.0 Å × 90.0 Å × 152.3 Å (117,636 atoms) for MmpL4 and MmpL5 using the CHARMM-GUI Membrane Builder webserver (http://www.charmm-gui.org/?doc=input/membrane) [52]. We then added 150 mM NaCl and extra neutralizing counter ions for the simulations. The Antechamber module of AmberTools was employed to generate parameters for Mbt by using the general AMBER force field (GAFF) [53,54]. The partial charges of Mbt were calculated using ab initio quantum chemistry at the HF/6-31G* level (GAUSSIAN 16 program) (Gaussian Inc., Wallingford). The RESP charge-fitting scheme was used to calculate partial charges on the atoms [55]. The tleap program was used to generate parameter and coordinate files using the ff14SB and Lipid17 force field for both the protein and lipids. The PMEMD.CUDA program implemented in AMBER18 (AMBER 2018, UCSF) was used to conduct MD simulations. The simulations were performed with periodic boundary conditions to produce isothermal-isobaric ensembles. Long-range electrostatics were calculated using the particle mesh Ewald (PME) method [56] with a 10 Å cutoff. In the simulations, we selected the heavy atoms of the 2-hydrophenyloxazoline-ring head group of Mbt to be guided towards the target position at the periplasmic lipid-binding site by the application of steering forces. The root mean square (RMS) distance between the current coordinates and the target structure was calculated at each timestep. The force on each selected atom was given by a gradient of potential as a function of the RMS values. The systems went through energy minimization, heating, and 5 ns equilibrium MD simulations. Following this, an additional 200 ns of unrestrained production MD simulations were conducted. TMD simulations were then performed based on the coordinates obtained after the production MD. A value of 500 kcal/mol/Å^2^ was used as an elastic constant for TMD forces during the simulations.

## Supporting information

S1 Table MmpL4 and MmpL5 cryo-EM data collection, processing, and refinement statistics. (PDF)

S1 Fig Phylogenetic analysis of MmpL proteins and the topologies of subclasses I and II. (A) Guide tree of MmpL proteins reveals 2 distinct subclasses. The Guide tree was calculated from the Uniprot website (https://www.uniprot.org/). (B) Membrane topologies of subclasses I and II of MmpL proteins. The PD1, PD2, PD3, and CD domains are labeled.
(TIF)

S2 Fig MmpL4 Data processing. (A) Data processing workflow of MmpL4. Side view of the MmpL4 cryo-EM map (contour level of 0.25). (B) Representative 2D classes of MmpL4. (C) Gold-Standard Fourier shell correlation (GS-FSC) curve of MmpL4. (D) Representative local cryo-EM map of MmpL4 (TM, left; PD1, middle; PD2, right). (E) Visual representation of the MmpL4 protein mask.
(TIF)

S3 Fig Structural comparison of MmpL3 and MmpL4. (A) Superimposition of the MmpL3 and MmpL4 structures. This superimposition is genetated by overlaying the structure of MmpL4 to that of MmpL3 (PDB ID: 7K8B) and the RMSD was calculated to be 5.9 Å (for 673 Cα atoms). (B) Superimposition of the structural elements of the N-terminal and C-terminal halves of MmpL4. This superimposition results in a high RMSD of 2.6 Å (for 294 Cα atoms).
(TIF)

S4 Fig The MmpL4 and MmpL5 proteins. (A) SEC trace and SDS-PAGE of purified MmpL4. The trace and gel image indicate that purified MmpL4 is a full-length protein. (B) SEC trace and SDS-PAGE of purified MmpL5. The trace and gel image indicate that purified MmpL5 is a full-length protein. (C) Low threshold cryo-EM map of MmpL4. The contour level was set to 0.04. (D) Low threshold cryo-EM map of MmpL5. The contour level was set to 0.04.
(TIF)

S5 Fig MmpL5 Data processing. (A) Data processing workflow of MmpL5. Side view of the MmpL5 cryo-EM map (contour level of 0.25). (B) Representative 2D classes of MmpL5. (C) Gold-Standard Fourier shell correlation (GS-FSC) curve of MmpL4. (D) Representative local cryo-EM map of MmpL5 (TM, left; PD1, middle; PD2, right). (E) Visual representation of the MmpL5 protein mask.
(TIF)

S6 Fig The MmpL4 channels. The MmpL4 protein forms 2 channels. One of the channels spans the pocket surrounded by TMs 7–10 at the outer leaflet of the cytoplasmic membrane and the cavity generated between subdomains PD1 and PD2 in the periplasm (red channel). The other channel spans the pocket surrounded by TMs 1–4 at the outer leaflet of the cytoplasmic membrane and the cavity generated between subdomains PD1 and PD2 in the periplasm (wheat channel). These channels were calculated using the program CAVER (https//www.caver.cz/).
(TIF)

S7 Fig Proton-relay networks. (A) The proton-relay network of MmpL3. Depicted are the conserved residues D256, Y257, D645, and Y646 thought to be important for proton transfer and energy coupling. (B) The proton-relay network of MmpL4. Depicted are the conserved residues D278, Y279, D851, and Y852 thought to be important for proton transfer and energy coupling. (C) The proton-relay network of MmpL5. Conserved residues D278, Y279, D851, and Y852, residues thought to be important for proton transfer and energy coupling, are highlighted.
(TIF)

S8 Fig Docking of Cmb and Bed to the structures of MmpL4 and MmpL5. (A) The predicted Cmb-binding site of MmpL4. The docked Cmb molecule is shown as yellow sticks (at the binding site located in the outer leaflet of the cytoplasmic membrane). The predicted binding affinity is calculated to be −7.0 kcal/mol. (B) The predicted Bed-binding site of MmpL4. The docked Bed molecule is shown as red sticks (at the binding site located in the outer leaflet of the cytoplasmic membrane). The predicted binding affinity is calculated to be −7.1 kcal/mol. (C) The predicted Cmb-binding sites of MmpL5. The docked Cmb molecules are shown as green sticks (with a predicted binding affinity of −6.9 kcal/mol at the binding site located in the outer leaflet of the cytoplasmic membrane) and blue sticks (with a predicted binding affinity of −7.4 kcal/mol at the binding site located in periplasmic domain). (D) The predicted Bed-binding site of MmpL5. The docked Bed molecule is shown as cyan sticks (at the binding site located in the outer leaflet of the cytoplasmic membrane). The predicted binding affinity is calculated to be −8.4 kcal/mol.
(TIF)

S9 Fig Proposed mechanism for Mbt translocation via MmpL4 or MmpL5. This schematic diagram indicates that the MmpL4 or MmpL5 transporter is capable of picking up an Mbt molecule from the outer leaflet of the cytoplasmic membrane. This Mbt siderophore will shuttle through the channel formed by MmpL4 or MmpL5 and reach the periplasmic Mbt-binding site for export.
(TIF)

S1 Raw Images SDS-PAGE uncropped gel images of purified MmpL4 and MmpL5. (TIFF)

## Abbreviations

cryo-EM: cryo-electron microscopy
CTF: contrast transfer function
EDTA: ethylenediaminetetraacetic acid
FSC: Fourier shell correlation
GAFF: general AMBER force field
LMNG: lauryl maltose neopentyl glycol
MA: mycolic acid
mAGP: mycolyl arabinogalactan peptidoglycan
Mbt: mycobactin
MD: molecular dynamics
MDR: multidrug-resistant
MmpL: mycobacterial membrane protein large
Mtb: *Mycobacterium tuberculosis*
MWE: mycolate wax ester
PDIM: phthiocerol dimycocerosate
PE: phosphatidylethanolamine
PME: particle mesh Ewald
PMF: proton motive force
PMSF: phenylmethylsulfonyl fluoride
RND: resistance-nodulation cell division
RMSD: root mean square deviation
SEC: size exclusion chromatography
SL: sulfolipid
TB: tuberculosis
TDM: trehalose dimycolate
TMD: targeted molecular dynamics
TMM: trehalose monomycolate
