# Supplementary material for: Structures of the mycobacterial MmpL4 and MmpL5 transporters provide insights into their role in siderophore export and iron acquisition
Source: PLoS Biol. 2024 Oct 18;22(10):e3002874. doi: 10.1371/journal.pbio.3002874 (PMC11524445; doi:10.1371/journal.pbio.3002874)
Supplement: S1 Table — (PDF) [file pbio.3002874.s001.pdf]

S1 Table. MmpL4 and MmpL5 cryo-EM data collection and refinement statistics.

Data collection	MmpL4		MmpL5	
Magnification	81,000	81,000	81,000	81,000
Voltage (kV)	300	300	300	300
Electron Microscope	Krios-GIF-K3	Krios-GIF-K3	Krios-GIF-K3	Krios-GIF-K3
Defocus (um)	-0.8 to -1.5	-0.8 to -1.5	-0.8 to -1.5	-0.8 to -1.5
Energy filter width (eV)	20	20	20	20
Pixel size (Å)	1.07 (0.535)	1.07 (0.535)	1.07 (0.535)	1.07 (0.535)
Total dose (e^-/ $Å^2$)	40.2	40.4	40.0	39.4
Number of frames	45	44	36	40
Number of micrographs	1,123	2,243	355	1,315
Number of initial particles	4,352,328		1,648,768	
Refinement				
Number of total particles	78,169		61,169	
GS-FSC Resolution (0.143, Å)[a]	2.95		3.00	
Model composition				
Chains	1		1	
Protein residues	734		726	
r.m.s.d.				
Bond lengths (Å)	0.004 (0)		0.003 (0)	
Bond angles (°)	0.480 (4)		0.465 (3)	
Validation				
MolProbity score	1.73		1.48	
Clash score	6.72		7.58	
Ramachandran plot				
Favored (%)	96.16		99.31	
Allowed (%)	3.84		0.69	
Disallowed (%)	0.00		0.00	
CC Mask	0.87		0.84	
