# Supplementary figures and images for: Structures of the mycobacterial MmpL4 and MmpL5 transporters provide insights into their role in siderophore export and iron acquisition
Source: PLoS Biol. 2024 Oct 18;22(10):e3002874. doi: 10.1371/journal.pbio.3002874 (PMC11524445; doi:10.1371/journal.pbio.3002874)

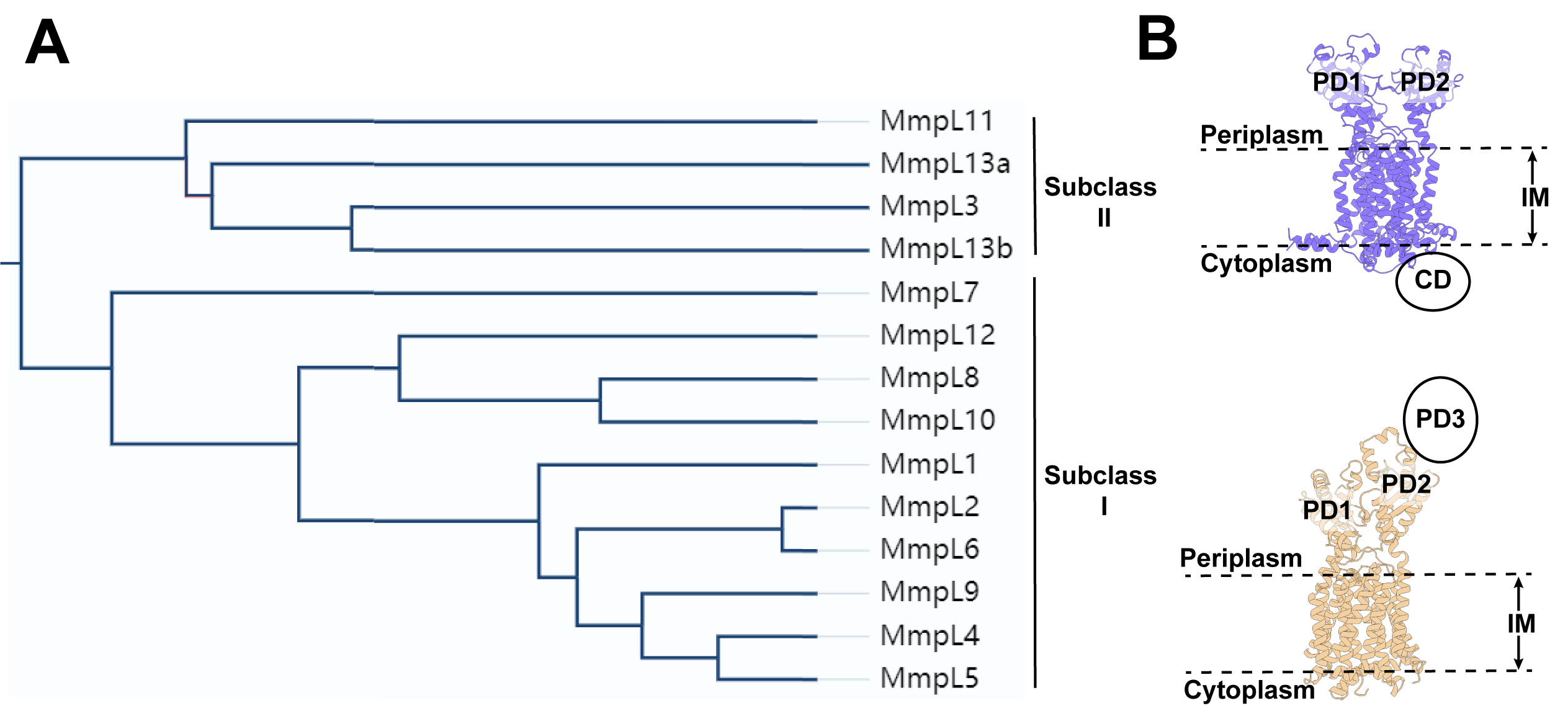

Supplement: S1 Fig — (A) Guide tree of MmpL proteins reveals 2 distinct subclasses. The Guide tree was calculated from the Uniprot website (https://www.uniprot.org/). (B) Membrane topologies of subclasses I and II of MmpL proteins. The PD1, PD2, PD3, and CD domains are labeled. (TIF) [file pbio.3002874.s002.tif]

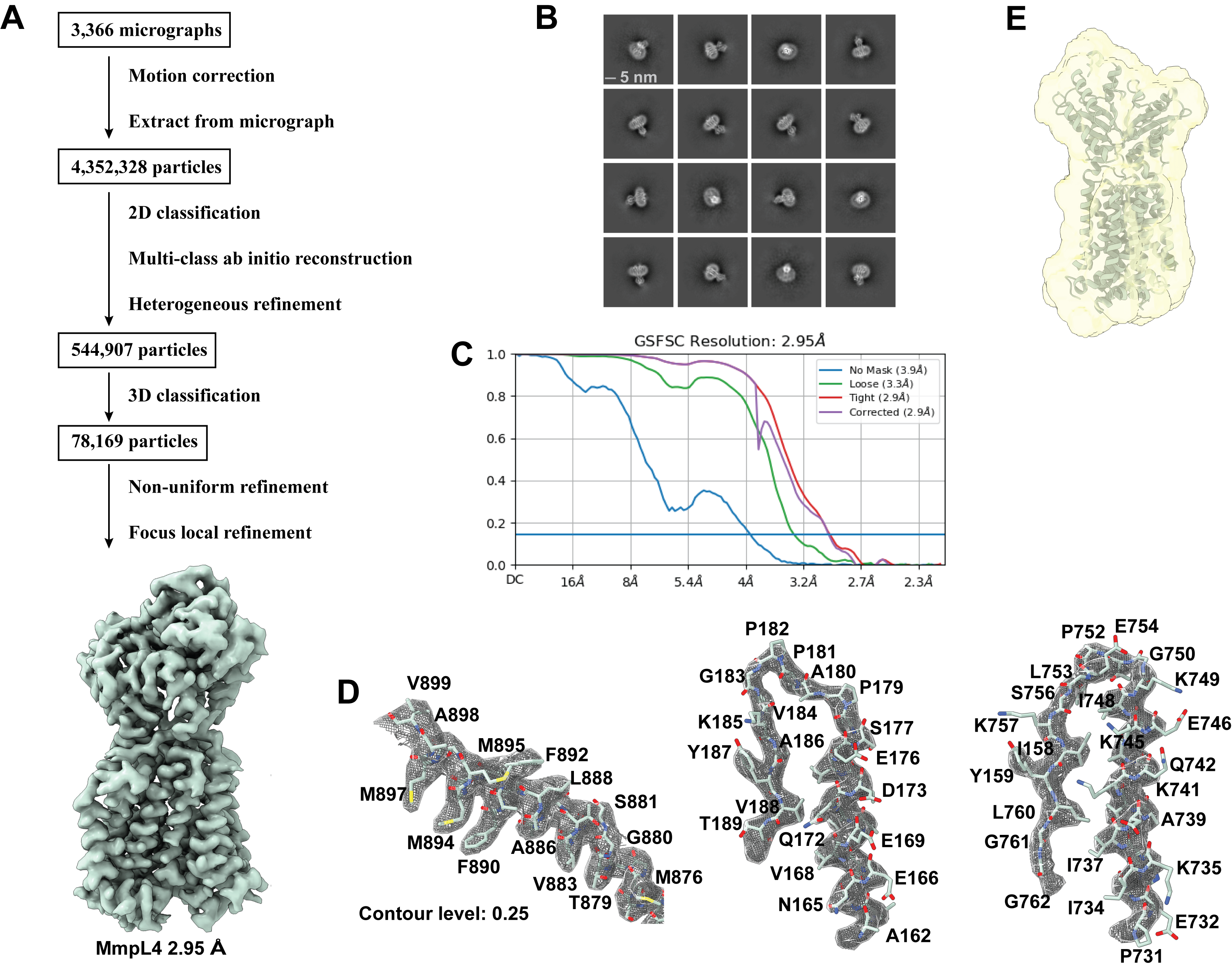

Supplement: S2 Fig — (A) Data processing workflow of MmpL4. Side view of the MmpL4 cryo-EM map (contour level of 0.25). (B) Representative 2D classes of MmpL4. (C) Gold-Standard Fourier shell correlation (GS-FSC) curve of MmpL4. (D) Representative local cryo-EM map of MmpL4 (TM, left; PD1, middle; PD2, right). (E) Visual representation of the MmpL4 protein mask. (TIF) [file pbio.3002874.s003.tif]

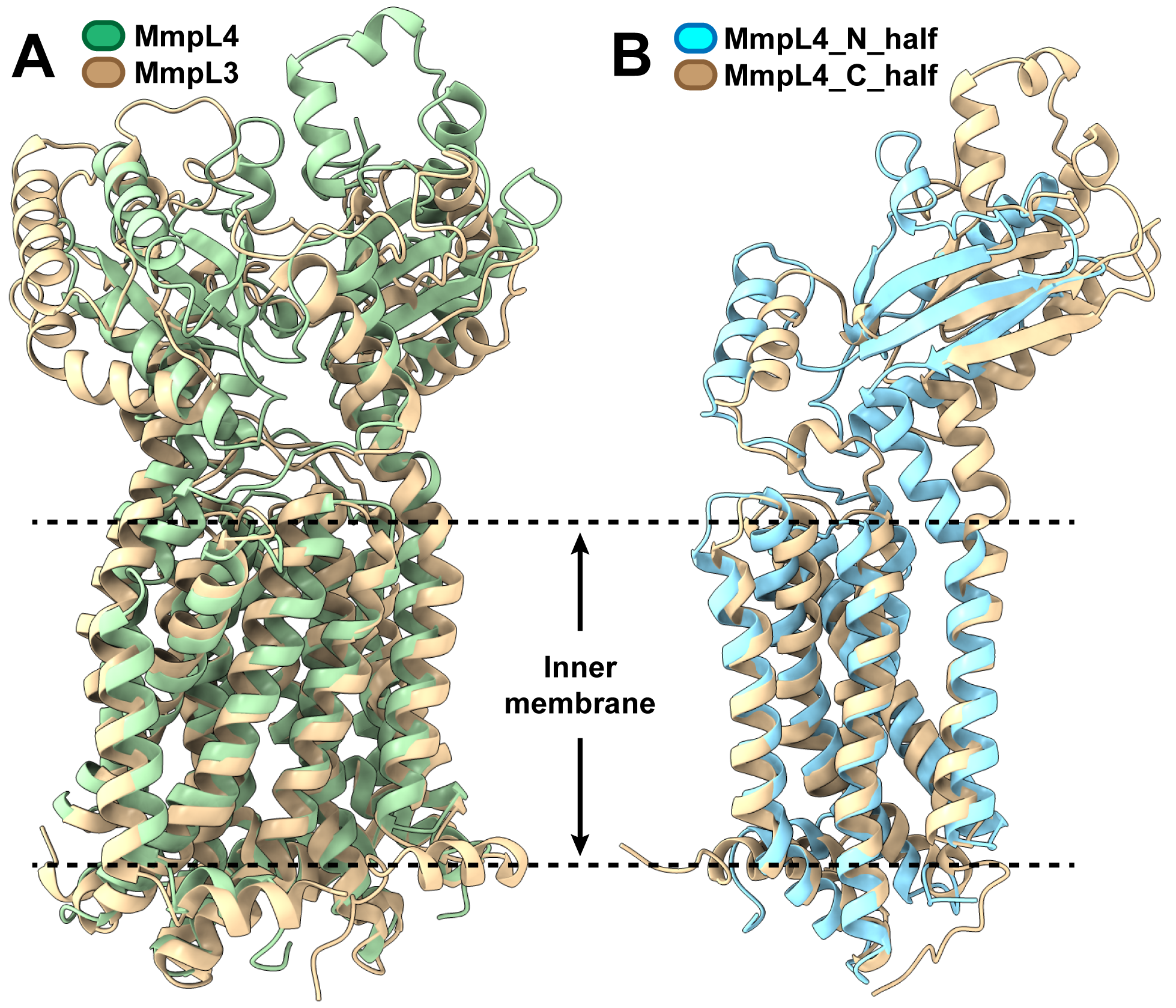

Supplement: S3 Fig — (A) Superimposition of the MmpL3 and MmpL4 structures. This superimposition is genetated by overlaying the structure of MmpL4 to that of MmpL3 (PDB ID: 7K8B) and the RMSD was calculated to be 5.9 Å (for 673 Cα atoms). (B) Superimposition of the structural elements of the N-terminal and C-terminal halves of MmpL4. This superimposition results in a high RMSD of 2.6 Å (for 294 Cα atoms). (TIF) [file pbio.3002874.s004.tif]

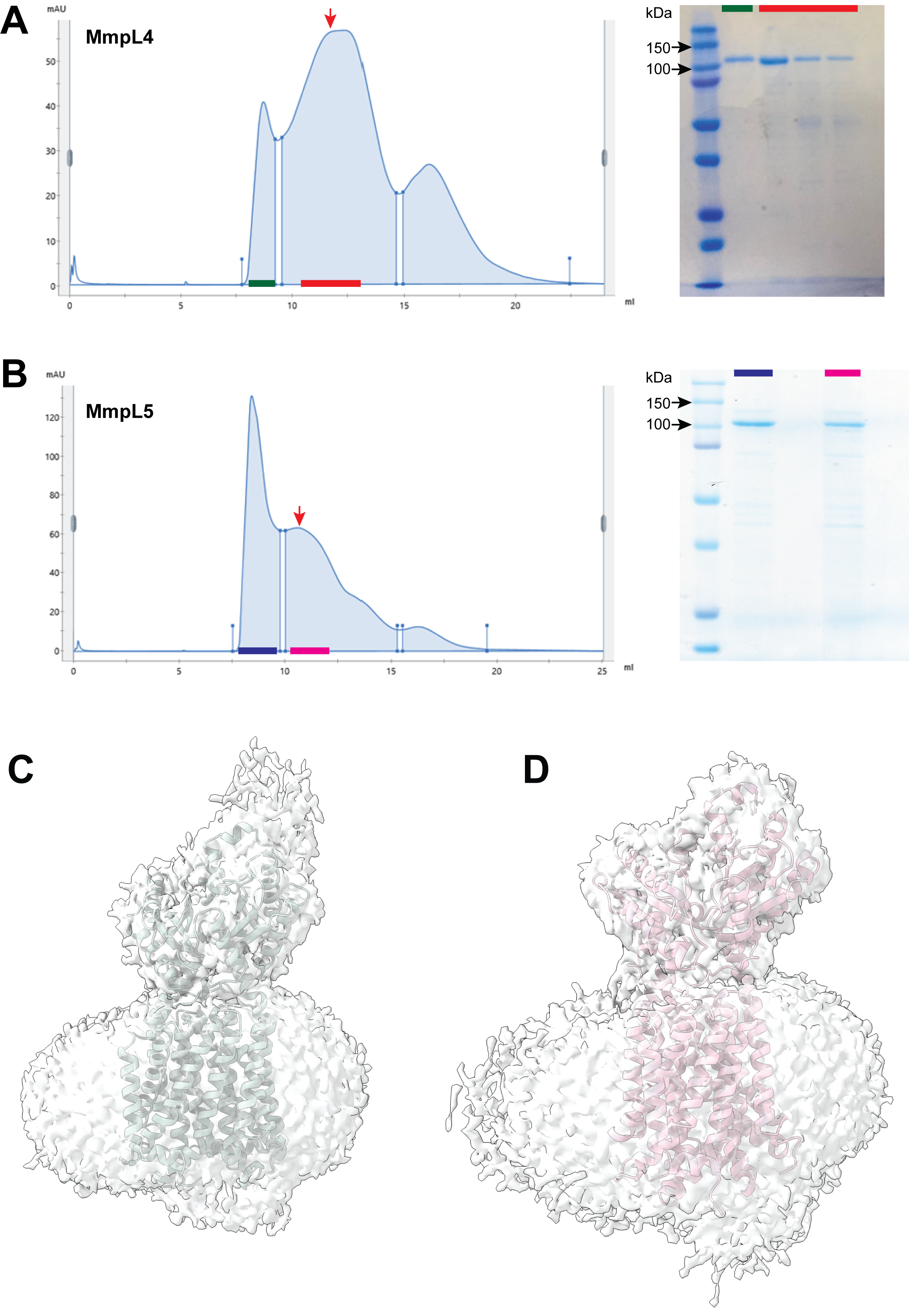

Supplement: S4 Fig — (A) SEC trace and SDS-PAGE of purified MmpL4. The trace and gel image indicate that purified MmpL4 is a full-length protein. (B) SEC trace and SDS-PAGE of purified MmpL5. The trace and gel image indicate that purified MmpL5 is a full-length protein. (C) Low threshold cryo-EM map of MmpL4. The contour level was set to 0.04. (D) Low threshold cryo-EM map of MmpL5. The contour level was set to 0.04. (TIF) [file pbio.3002874.s005.tif]

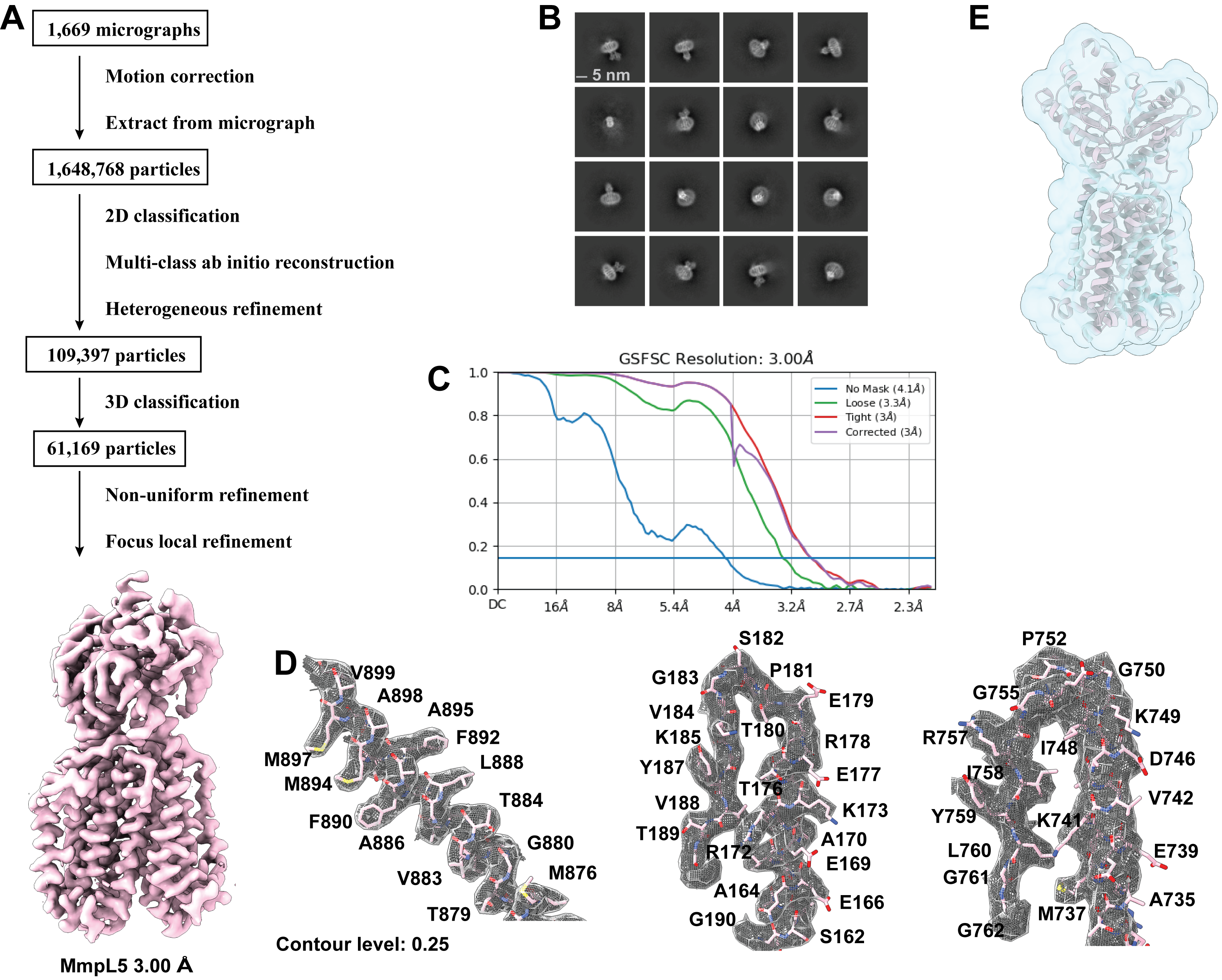

Supplement: S5 Fig — (A) Data processing workflow of MmpL5. Side view of the MmpL5 cryo-EM map (contour level of 0.25). (B) Representative 2D classes of MmpL5. (C) Gold-Standard Fourier shell correlation (GS-FSC) curve of MmpL4. (D) Representative local cryo-EM map of MmpL5 (TM, left; PD1, middle; PD2, right). (E) Visual representation of the MmpL5 protein mask. (TIF) [file pbio.3002874.s006.tif]

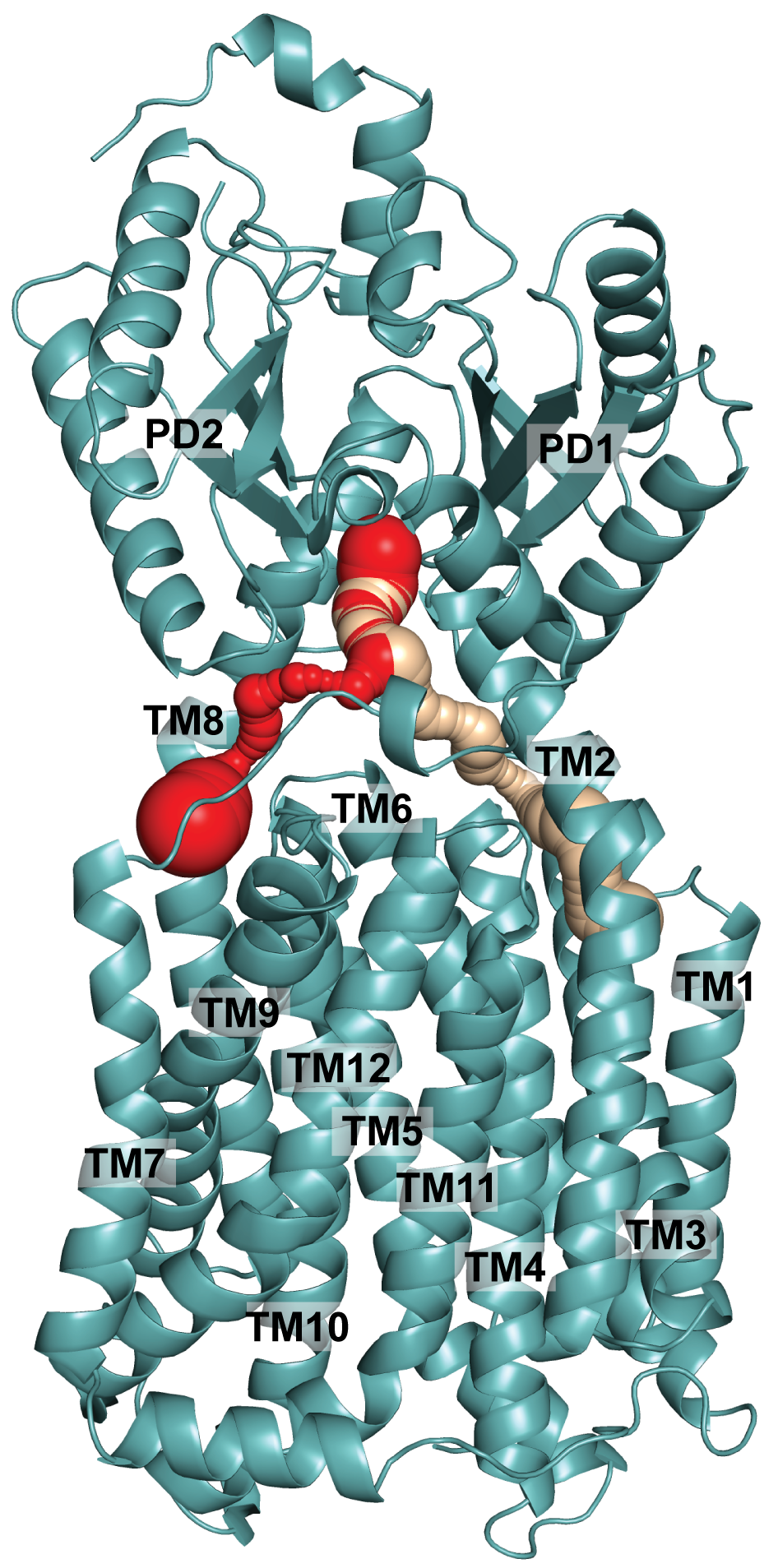

Supplement: S6 Fig — The MmpL4 protein forms 2 channels. One of the channels spans the pocket surrounded by TMs 7–10 at the outer leaflet of the cytoplasmic membrane and the cavity generated between subdomains PD1 and PD2 in the periplasm (red channel). The other channel spans the pocket surrounded by TMs 1–4 at the outer leaflet of the cytoplasmic membrane and the cavity generated between subdomains PD1 and PD2 in the periplasm (wheat channel). These channels were calculated using the program CAVER (https//www.caver.cz/). (TIF) [file pbio.3002874.s007.tif]

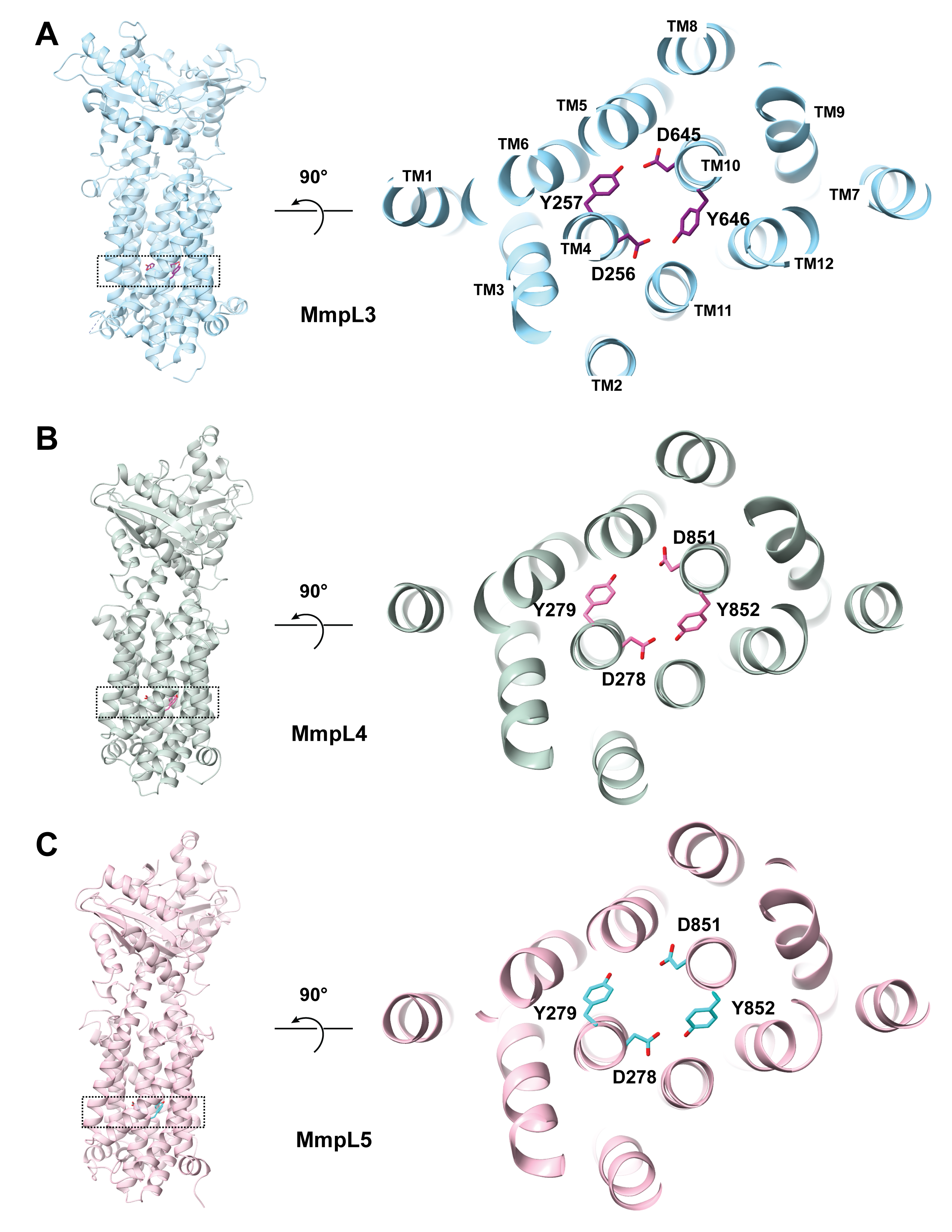

Supplement: S7 Fig — (A) The proton-relay network of MmpL3. Depicted are the conserved residues D256, Y257, D645, and Y646 thought to be important for proton transfer and energy coupling. (B) The proton-relay network of MmpL4. Depicted are the conserved residues D278, Y279, D851, and Y852 thought to be important for proton transfer and energy coupling. (C) The proton-relay network of MmpL5. Conserved residues D278, Y279, D851, and Y852, residues thought to be important for proton transfer and energy coupling, are highlighted. (TIF) [file pbio.3002874.s008.tif]

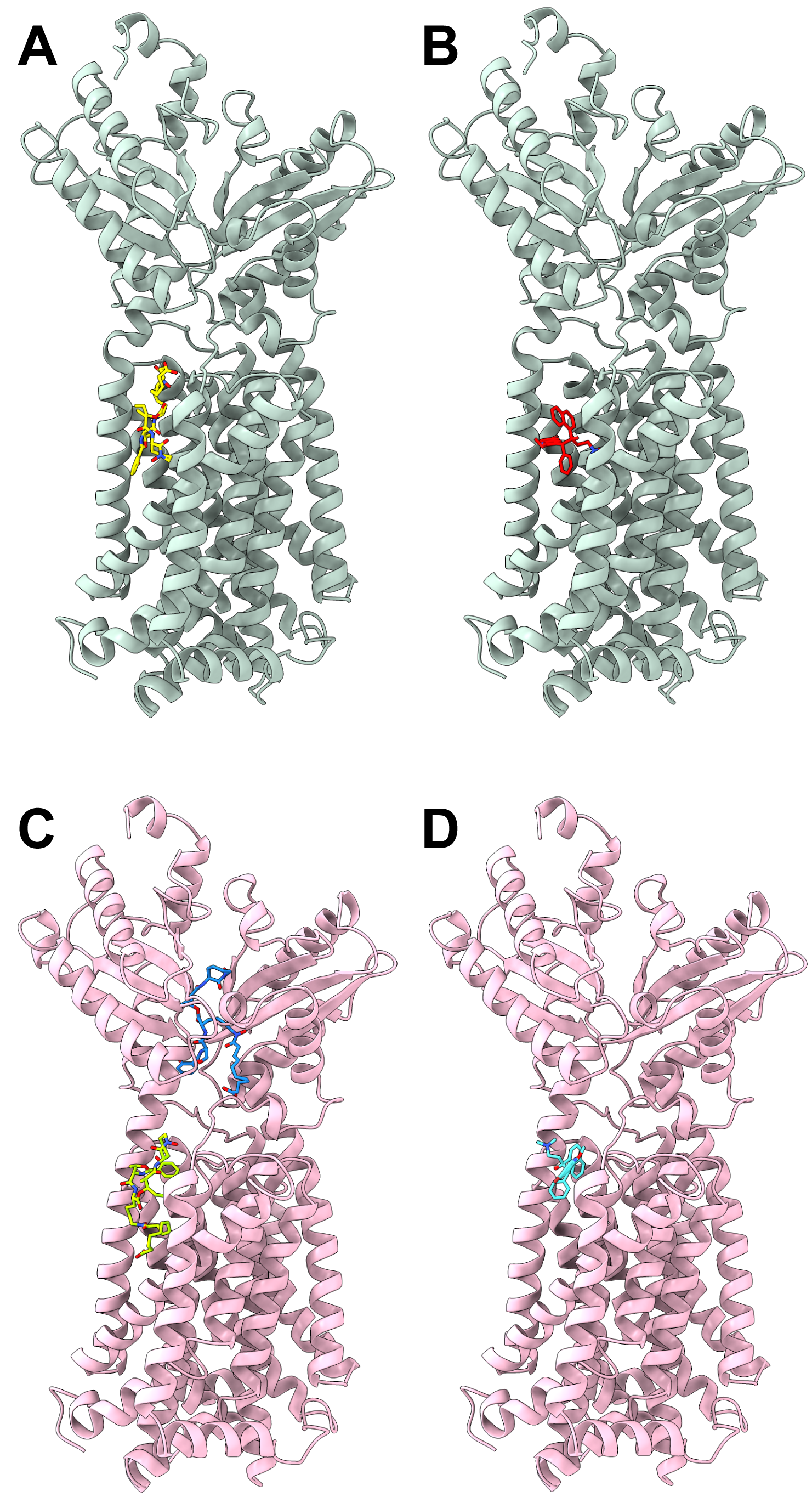

Supplement: S8 Fig — (A) The predicted Cmb-binding site of MmpL4. The docked Cmb molecule is shown as yellow sticks (at the binding site located in the outer leaflet of the cytoplasmic membrane). The predicted binding affinity is calculated to be −7.0 kcal/mol. (B) The predicted Bed-binding site of MmpL4. The docked Bed molecule is shown as red sticks (at the binding site located in the outer leaflet of the cytoplasmic membrane). The predicted binding affinity is calculated to be −7.1 kcal/mol. (C) The predicted Cmb-binding sites of MmpL5. The docked Cmb molecules are shown as green sticks (with a predicted binding affinity of −6.9 kcal/mol at the binding site located in the outer leaflet of the cytoplasmic membrane) and blue sticks (with a predicted binding affinity of −7.4 kcal/mol at the binding site located in periplasmic domain). (D) The predicted Bed-binding site of MmpL5. The docked Bed molecule is shown as cyan sticks (at the binding site located in the outer leaflet of the cytoplasmic membrane). The predicted binding affinity is calculated to be −8.4 kcal/mol. (TIF) [file pbio.3002874.s009.tif]

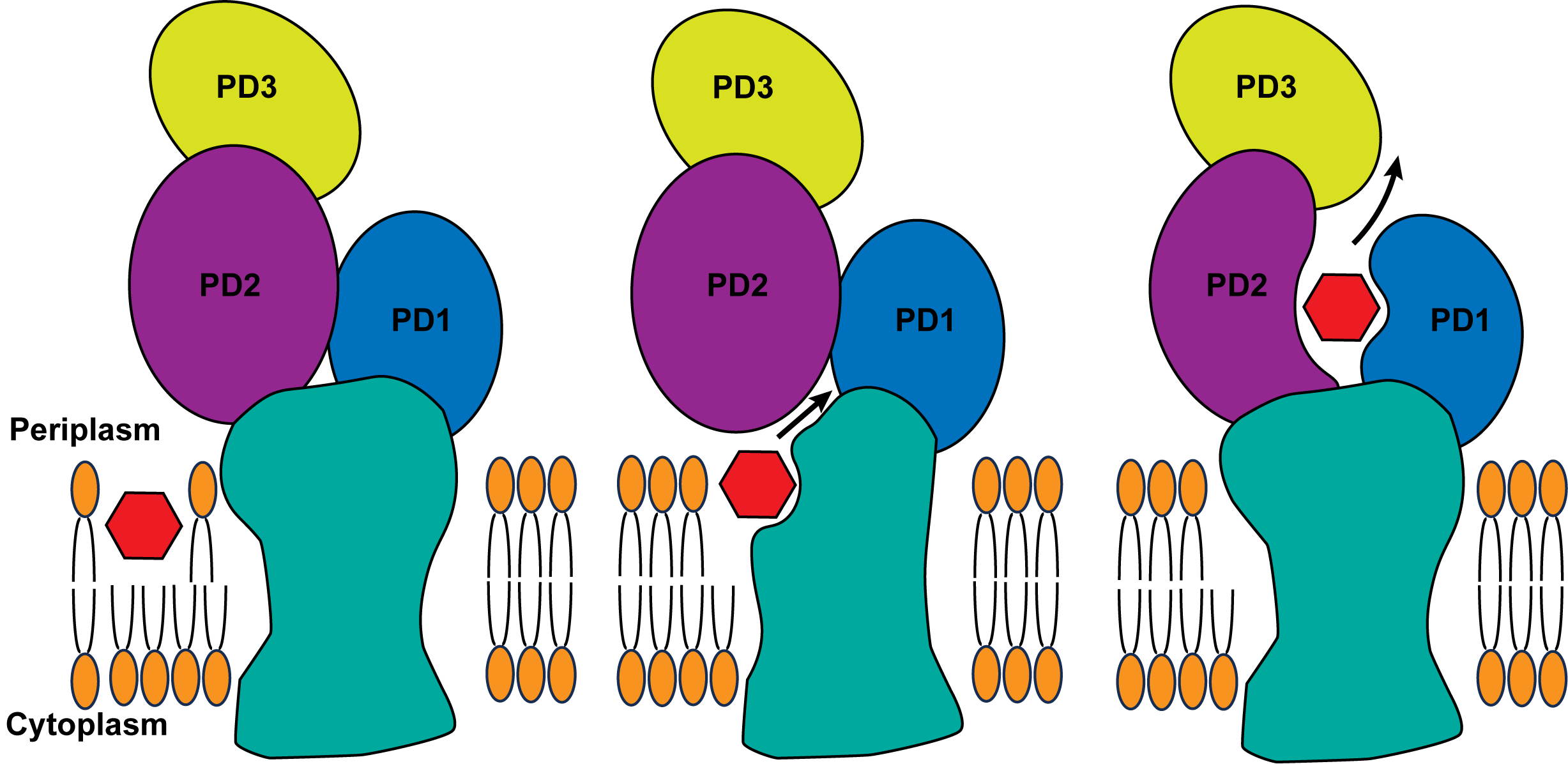

Supplement: S9 Fig — This schematic diagram indicates that the MmpL4 or MmpL5 transporter is capable of picking up an Mbt molecule from the outer leaflet of the cytoplasmic membrane. This Mbt siderophore will shuttle through the channel formed by MmpL4 or MmpL5 and reach the periplasmic Mbt-binding site for export. (TIF) [file pbio.3002874.s010.tif]

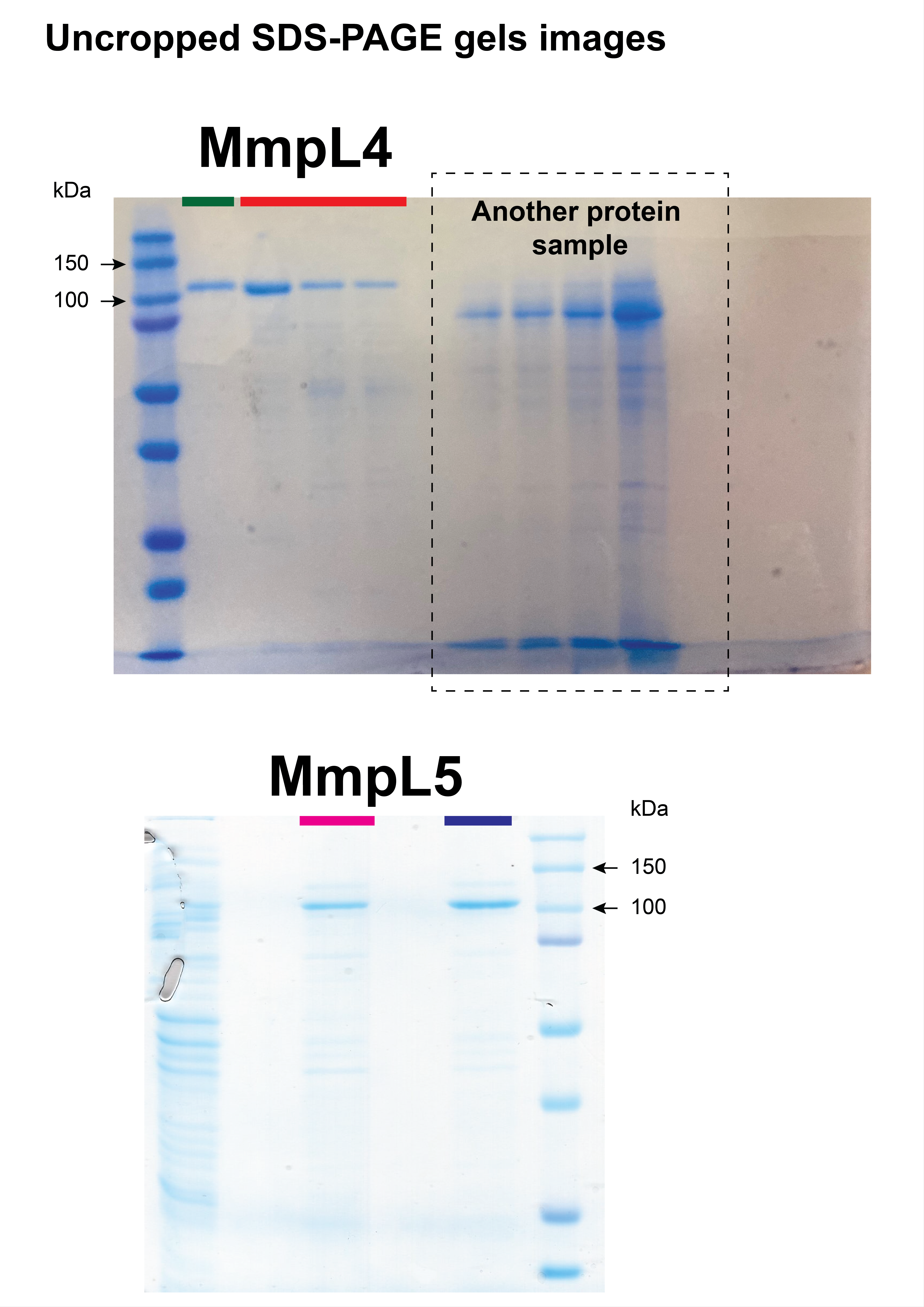

Supplement: S1 Raw Images — (TIFF) [file pbio.3002874.s011.tiff]
